# A Simple Protocol for the Determination of Lysostaphin Enzymatic Activity

**DOI:** 10.3390/antibiotics9120917

**Published:** 2020-12-17

**Authors:** Alexander V. Grishin, Svetlana V. Konstantinova, Irina V. Vasina, Nikita V. Shestak, Anna S. Karyagina, Vladimir G. Lunin

**Affiliations:** 1 N.F. Gamaleya National Research Center for Epidemiology and Microbiology, Ministry of Health of the Russian Federation, 123098 Moscow, Russia; konstantinova@gamaleya.org (S.V.K.); vasina@gamaleya.org (I.V.V.); nikita1305@mail.ru (N.V.S.); karyagina@gamaleya.org (A.S.K.); lunin@gamaleya.org (V.G.L.); 2 All-Russia Research Institute of Agricultural Biotechnology, Russian Academy of Sciences, 127550 Moscow, Russia; 3 Faculty of Bioengineering and Bioinformatics, M.V. Lomonosov Moscow State University, 119234 Moscow, Russia; 4 A.N. Belozersky Institute of Physical and Chemical Biology, M.V. Lomonosov Moscow State University, 119992 Moscow, Russia

**Keywords:** lysostaphin, pentaglycine, enzymatic activity, catalytic efficiency, protocol, assay, lysin, *Staphylococcus aureus*

## Abstract

Antibacterial lysins are enzymes that hydrolyze bacterial peptidoglycan, which results in the rapid death of bacterial cells due to osmotic lysis. Lysostaphin is one of the most potent and well-studied lysins active against important nosocomial pathogen *Staphylococcus aureus*. Similarly to most other lysins, lysostaphin is composed of enzymatic and peptidoglycan-binding domains, and both domains influence its antibacterial activity. It is thus desirable to be able to study the activity of both domains independently. Lysostaphin cleaves pentaglycine cross-bridges within the staphylococcal peptidoglycan. Here, we report the protocol to study the catalytic activity of lysostaphin on the isolated pentaglycine peptide that is based on the chromogenic reaction of peptide amino groups with ninhydrin. Unlike previously reported assays, this protocol does not require in-house chemical synthesis or specialized equipment and can be readily performed in most laboratories. We demonstrate the use of this protocol to study the effect of EDTA treatment on the lysostaphin enzymatic activity. We further used this protocol to determine the catalytic efficiency of lysostaphin on the isolated pentaglycine and compared it to the apparent catalytic efficiency on the whole staphylococcal cells. These results highlight the relative impact of enzymatic and peptidoglycan-binding domains of lysostaphin on its bacteriolytic activity.

## 1. Introduction

Lysostaphin is a glycyl-glycine endopeptidase that possesses potent bactericidal activity towards the important nosocomial pathogen *Staphylococcus aureus* [1]. Lysostaphin is an archetypal example of a wide class of proteins called antibacterial lysins. These enzymes cleave various bonds within bacterial peptidoglycan, rendering bacterial cells vulnerable to osmotic lysis. Antibacterial lysins are effective against antibiotic-resistant bacterial strains and are promising candidates for the treatment of antibiotic-resistant infections [2]. Thus, simple and reliable methods for the accurate determination of their activity are required, both to compare the activity of different lysins and lysin variants and for quality control of lysin preparations.

Lysostaphin is a 27 kDa protein that consists of an *N*-terminal catalytic domain and a *C*-terminal peptidoglycan-binding domain. Upon binding to the peptidoglycan, lysostaphin cleaves pentaglycine cross-bridges that are specific to *S. aureus* peptidoglycan, which ultimately leads to the degradation of the cell wall, bacterial lysis, and death [1]. The activity of lysostaphin, as well as other antibacterial lysins, is usually determined by the so-called turbidity assay. A suspension of bacterial cells is prepared in the desired buffer, then lysostaphin is added, and the optical density of the suspension is measured at certain time points. As bacterial cells lyse under the action of lysostaphin, they no longer scatter light, and the optical density of the suspension decreases. Bacteriolytic activity is then expressed either as the decrease in optical density per minute in the linear region of the curve [3,4] or as the amount of enzyme required to reduce the optical density by a certain percentage after a defined period of time [4,5]. Although this method is simple, it involves handling living staphylococci, which is not always possible due to biosafety constraints. While killed staphylococci can be used, they produce results of poorer quality [6,7,8]. More importantly, this method only determines the cumulative bacteriolytic activity of lysostaphin, and the results are inevitably influenced by the binding of lysostaphin to the bacterial cells and the susceptibility of particular staphylococcal strain to osmotic lysis. A method to directly estimate the enzymatic activity of lysostaphin towards its pentaglycine substrate is thus desirable.

Several such methods have been proposed. In one approach, pentaglycine or pentaglycine-containing peptides can be tagged with a fluorophore and a quencher and used as a substrate in a Förster resonance energy transfer (FRET)-based pentaglycine cleavage assay [9,10]. A similar principle was utilized to create a recombinant protein with a pentaglycine spacer between a green fluorescent protein GFPuv and a fragment of colicin E9 chemically modified with a fluorescent dye [11]. Cleavage of this substrate can be monitored either by fluorescence spectroscopy or by separating the cleavage products on SDS-PAGE. Although these assays proved instrumental in the study of lysostaphin cleavage sequence specificity, they were restricted to very low substrate concentrations due to the solubility issues and the inner filter effect. In a different approach, the *N*-acetylation of hexaglycine peptide was used to protect the *N*-terminal amino group, and substrate hydrolysis was determined by spectrophotometry after a chromogenic reaction between 2,4,6-trinitrobenzenesulfonic acid (TNBS) and a newly exposed amino group of the hexaglycine cleavage product [12]. Alternatively, unmodified pentaglycine peptide can be used as a substrate, and the detection of the reaction products—di- and triglycine peptides—can be performed by ^1^H-NMR [13,14].

Unfortunately, all these protocols require specialized equipment and/or in-house chemical synthesis of the substrates. In this work, we propose a simple protocol for the determination of lysostaphin enzymatic activity that only requires inexpensive and easily accessible reagents and basic laboratory equipment. The protocol is based on the well-known chromogenic reaction between ninhydrin and N-terminal amino groups of amino acids and peptides.

## 2. Results and Discussion

### 2.1. General Protocol Description

The catalytic domain of lysostaphin cleaves pentaglycine peptide between second and third or third and fourth glycine residues [9], generating di- and triglycine peptides in either case. Since every di-, tri-, or pentaglycine molecule has an *N*-terminal amino group, the total amount of amino groups in the reaction mixture increases by one per every pentaglycine molecule cleaved. This increase in the number of amino groups can be conveniently detected by the chromogenic reaction with ninhydrin. The effect is further enhanced by the more efficient reaction of ninhydrin with shorter oligoglycine peptides.

The protocol steps are as follows:

- Prepare 5 mM pentaglycine stock solution in water by heating the suspension of pentaglycine powder at 99 °C for 20 min.
- Mix pentaglycine stock solution, desired buffer stock solution, and lysostaphin stock solution.
- Aliquot the reaction mixture into 20 μL aliquots in 0.5 mL Eppendorf tubes and incubate the aliquots at the temperature of choice.
- At selected time points, remove the aliquots and place them at −80 °C to stop the reaction and preserve the samples for subsequent analysis.
- Thaw the samples at 99 °C for 10 min, add 100 μL of 0.4% *w*/*v* ninhydrin in 80% DMSO/20% water mixture buffered at pH 7.5, and mix thoroughly.
- Incubate the samples at 85 °C for 15 min. The color of the samples should become blue.
- Cool the samples to room temperature, add 200 µL of water, and mix. The color of the samples should turn violet.
- Transfer 100 µL of each sample into the wells of a 96-well plate and measure the optical density at 595 nm using a microplate reader.

**Figure 1:**
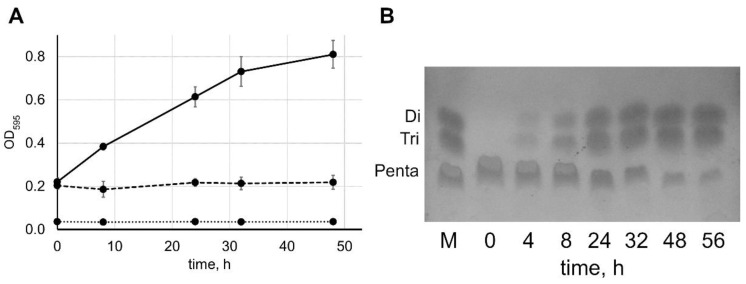
Hydrolysis of pentaglycine by lysostaphin monitored by the chromogenic reaction with ninhydrin and thin-layer chromatography. (**A**) We mixed 4 mM pentaglycine in 20 mM 4-(2-hydroxyethyl)-1-piperazineethanesulfonic acid (HEPES), pH 7.5, with 5 µM (solid line) lysostaphin, or without lysostaphin (dashed line); incubated the mixture at 37 °C for 0–48 h; and treated it with ninhydrin. The dotted line represents 5 µM lysostaphin alone (without pentaglycine) treated in the same way. Average results from six (5 µM lysostaphin), two (without lysostaphin), or one (without pentaglycine) independent experiments are shown; error bars represent standard deviation, and the OD_595_ readings without lysostaphin were equal to the OD_595_ of the empty wells. (**B**) We mixed 4 mM pentaglycine in 20 mM HEPES, pH 7.5, with 5 µM lysostaphin; incubated the mixture at 37 °C for 0–56 h; and separated the reaction products by thin-layer chromatography. M—marker (a mixture of 3 mM di-, tri-, and pentaglycine).

An exemplary plot of optical density vs. time is presented in Figure 1A. In the presence of lysostaphin, the optical density increased from 0.220 ± 0.020 (corresponding to the fully intact pentaglycine) to 0.810 ± 0.064 in 48 h. In contrast, the optical density did not change with time in the absence of lysostaphin (Figure 1A). Lysostaphin itself did not influence the color intensity—in the absence of pentaglycine, the optical density was equal to the optical density of the empty wells and did not change with time (Figure 1A). To verify that the increase in color intensity was indeed due to the cleavage of pentaglycine into di- and triglycine, we analyzed the lysostaphin-treated samples by thin-layer chromatography (Figure 1B and Figure S1). The amount of pentaglycine decreased and the amount of di- and triglycine increased as the reaction progressed, concurrently with the increase in the color intensity of ninhydrin-treated samples.

### 2.2. Reaction Speed Depends on the Concentrations of Lysostaphin and Pentaglycine

**Figure 2:**
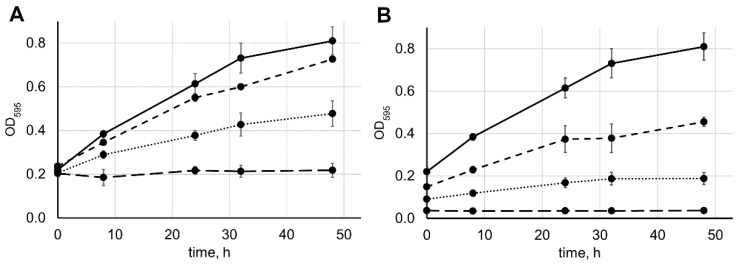
The dependence of pentaglycine cleavage rate on the concentrations of lysostaphin and pentaglycine. (**A**) We mixed 4 mM pentaglycine in 20 mM HEPES, pH 7.5, with 1 µM (dotted line), 3 µM (dashed line), or 5 µM (solid line) lysostaphin, or without lysostaphin (long-dash line); incubated the mixture at 37 °C for 0–48 h; and reacted the mixture with ninhydrin. Average results from six (5 µM lysostaphin, same as in Figure 1A), three (1 and 3 µM lysostaphin), or two (without lysostaphin, same as in Figure 1A) independent experiments are shown; error bars represent standard deviation. (**B**) We mixed 2 mM (dotted line), 3 mM (dashed line), 4 mM (solid line), or 0 mM (long-dash line) pentaglycine in 20 mM HEPES, pH 7.5, with 5 µM lysostaphin; incubated the mixture at 37 °C for 0–48 h; and reacted the mixture with ninhydrin. Average results from six (4 mM pentaglycine, same as in Figure 1A), three (2 and 3 mM pentaglycine), or one (without pentaglycine) independent experiments are shown; error bars represent standard deviation.

To further verify that the presented protocol captures the process of the pentaglycine cleavage by lysostaphin, we incubated pentaglycine with different concentrations of lysostaphin and monitored the color intensity of ninhydrin-treated samples. As shown in Figure 2A, higher concentrations of lysostaphin resulted in higher reaction speed. With 4 mM pentaglycine, the initial reaction rates during the first 8 h were 0.0204 ± 0.0035 ∆OD_595_ h^−1^ at 5 µM lysostaphin, 0.0176 ± 0.0070 ∆OD_595_ h^−1^ at 3 µM lysostaphin, and 0.0104 ± 0.0031 ∆OD_595_ h^−1^ at 1 µM lysostaphin (the difference between reaction rates at three lysostaphin concentrations was statistically significant, ANOVA *p*-value = 0.034). Next, we incubated 5 µM lysostaphin with different concentrations of pentaglycine (Figure 2B). As expected, the initial and the maximal color intensity, as well as the initial reaction rate, increased concomitantly with the increase in the starting pentaglycine concentration. The reaction rates during the first 8 h were 0.0036 ± 0.0007 ∆OD_595_ h^−1^ at 2 mM pentaglycine and 0.0099 ± 0.0006 ∆OD_595_ h^−1^ at 3 mM pentaglycine (the difference between reaction rates at the three pentaglycine concentrations was statistically significant, ANOVA *p*-value = 2.4 × 10^−5^).

The dependence of the initial reaction rate on the starting substrate concentration indicates that the substrate was not in excess. Unfortunately, achieving higher concentrations of pentaglycine was not possible due to its low solubility in water. Pentaglycine can be dissolved up to ≈10% *w*/*v* in concentrated formic acid. However, this approach results in the presence of exceedingly high amounts of formic acid salts when the stock solution of pentaglycine is diluted to the working concentration range and neutralized by the addition of a base. In contrast, our protocol allows for the use of arbitrary concentrations of salts and for investigation of the lysostaphin enzymatic activity under physiological conditions.

### 2.3. Comparison of the Enzymatic Activity of Lysostaphin Variants

The presented assay can be used to study the enzymatic activity of different lysostaphin variants. To demonstrate this, we treated lysostaphin with EDTA for 3 h at 37 °C to remove the catalytic zinc ion from the active center of the enzyme. Lysostaphin is a zinc-dependent peptidase, and removal of the zinc ion is expected to negatively affect its catalytic activity. Lysostaphin treated in the same way but without EDTA was used as a control. We then incubated pentaglycine with the control and EDTA-treated lysostaphin variants. The optical density for both control and EDTA-treated variants was 0.20 ± 0.01 at the 0 h time point. However, after 30 h of incubation, the optical density of control samples reached 0.55 ± 0.07, while the optical density of the EDTA-treated samples only increased to 0.23 ± 0.01 (*n* = 3 independent experiments, *t*-test *p*-value = 0.016) (Figure S2), indicating that treatment with EDTA almost completely abolished the enzymatic activity of lysostaphin.

### 2.4. Calculation of the Lysostaphin Catalytic Parameters

**Figure 3:**
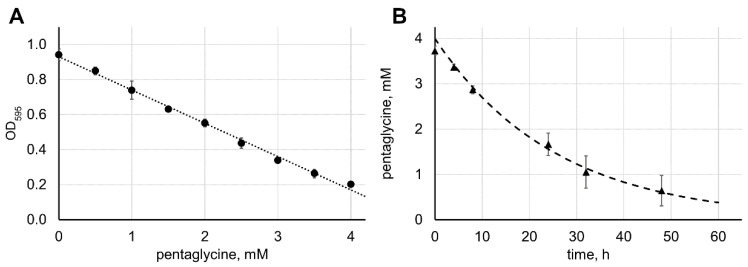
Derivation of lysostaphin catalytic parameters. (**A**) Di-, tri-, and pentaglycine were mixed, aliquoted, frozen, thawed, and treated with ninhydrin identically to lysostaphin-treated samples. To simulate the process of pentaglycine hydrolysis, we chose the concentrations of di- and triglycine so that at 4 mM pentaglycine, no di- or triglycine were added; at 3 mM pentaglycine, 1 mM each of di- and triglycine were added; etc. At 0 mM pentaglycine, the mixture contained 4 mM each of di- and triglycine. The average results from three independent experiments are shown; error bars represent standard deviation. (**B**) The calibration curve from panel A was used to convert the optical density values of the reaction of 4 mM pentaglycine with 5 µM lysostaphin (Figure 1A) into the pentaglycine concentrations and fitted with the simplified Michaelis–Menten equation (dashed line).

The protocol can further be used to derive the catalytic efficiency of lysostaphin by fitting the Michaelis–Menten equation into the kinetics of pentaglycine cleavage. To convert the optical density into the concentration of pentaglycine, we created a calibration curve by mixing appropriate amounts of pentaglycine, diglycine, and triglycine and treating them with ninhydrin (Figure 3A).

As seen in Figure 3A, the resulting calibration curve demonstrates a linear growth of the optical density with the decreasing concentration of pentaglycine and increasing concentrations of di- and triglycine on the whole range of the concentrations used in the assay. Thus, the optical density values could be straightforwardly converted into the pentaglycine concentrations (Figure 3B). To derive the catalytic parameters *k_cat_* and *K_M_* of lysostaphin, we tried to fit the Michaelis–Menten equation $\frac{d\left[S\right]}{dt}=-k_{cat}E_{0}\frac{\left[S\right]}{\left[S\right]+K_{M}}$ into the pentaglycine concentration vs. time data using nonlinear regression. However, the fitting gave unrealistic values of the *k_cat_* and *K_M_* parameters. This was likely due to the *K_M_* value of pentaglycine being much higher than the concentrations used in the assay, that is, *K_M_* ≫ [S]. In this case, the term $\frac{\left[S\right]}{\left[S\right]+K_{M}}$ becomes indistinguishable from $\frac{\left[S\right]}{K_{M}}$, and the Michaelis–Menten equation reduces to $\frac{d\left[S\right]}{dt}=-E_{0}\frac{k_{cat}}{K_{M}}\left[S\right]$. In this equation, it is impossible to separate individual parameters *k*_cat_ and *K_M_*, and only the catalytic efficiency parameter *k_cat_*/*K_M_* can be obtained. Fitting this equation into the experimental data yielded a catalytic efficiency of 2.2 M^−1^ s^−1^ (Figure 3B).

### 2.5. Comparison of Enzymatic and Bacteriolytic Activities of Lysostaphin

**Figure 4:**
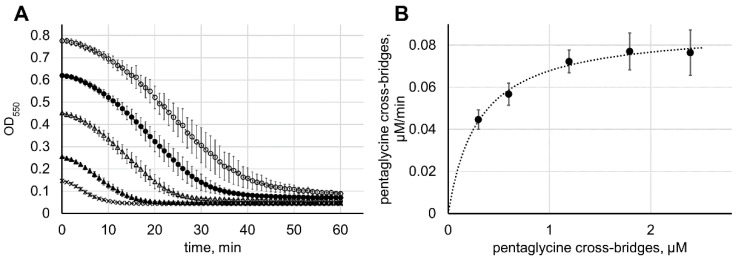
Derivation of apparent catalytic parameters of lysostaphin on pentaglycine incorporated into the cell wall. (**A**) Staphylococcal cells at different initial densities (open circles—8.0 McFarland, filled circles—6.0 McFarland, open triangles—4.0 McFarland, filled triangles—2.0 McFarland, crosses—1.0 McFarland) were mixed with 30 nM lysostaphin, incubated at 37 °C with constant shaking, and the optical density was measured at 1 min intervals at λ = 550 nm. The average results from three independent experiments are shown; error bars represent standard deviation. (**B**) The rate of decrease of staphylococcal cell suspension turbidity at different starting cell densities was calculated by approximating the curves (panel (**A**)) with five parameters logistic equation and taking the first derivative at the inflection point. These rates were converted to the rates of pentaglycine cross-bridge hydrolysis, plotted against initial pentaglycine cross-bridge concentration, and fitted with the Michaelis–Menten equation (dotted line). The average pentaglycine cross-bridge hydrolysis rates from three independent experiments are shown (filled circles); error bars represent standard deviation.

Despite the slow rate of hydrolysis of isolated pentaglycine peptide, lysostaphin rapidly lyses intact staphylococcal cells even in extremely low concentrations. To estimate the catalytic efficiency of lysostaphin towards pentaglycine incorporated into the staphylococcal cell wall, we measured the rate of decrease in the turbidity of staphylococcal cell suspensions with different starting cell density under the action of lysostaphin (Figure 4A). The turbidity can be converted into the concentration of bacterial cells, which in turn can be converted into the concentration of pentaglycine cross-bridges if the amount of the cross-bridges per cell is known. Assuming the direct relationship between the amount of hydrolyzed pentaglycine and the cell lysis, the rate of turbidity decrease can be converted into the rate of pentaglycine cross-bridge cleavage in the same way. Plotting the starting pentaglycine cross-bridge concentration vs. the pentaglycine cross-bridge cleavage rate, one can obtain the apparent catalytic parameters for the reaction, and taking ≈6 × 10^5^ pentaglycine bridges per staphylococcal cell [15], we estimated the apparent catalytic parameters of lysostaphin towards the pentaglycine incorporated into the cell wall to be *k_cat_* = 0.05 s^−1^, *K_M_* = 301 nM, *k_cat_*/*K_M_* = 1.6 × 10^5^ M^−1^ s^−1^ (Figure 4B). This represents almost five orders of magnitude increase compared to the catalytic efficiency towards the isolated pentaglycine peptide.

This dramatic increase in the catalytic efficiency can likely be attributed to the cell wall binding domain of lysostaphin. In the suspension of staphylococcal cells, the pentaglycine peptides were not uniformly distributed throughout the reaction volume but were partitioned to the cell wall, creating the zones of locally increased concentration. The cell wall binding domain targeted lysostaphin into these zones of high local substrate concentrations, effectively increasing the apparent catalytic efficiency of the enzyme. In principle, this accumulation of the substrate and the enzyme within a restricted volume of the cell wall should only affect the apparent *K_M_*, but not *k_cat_*. Indeed, the apparent *k_cat_* of lysostaphin calculated from the rate of cell lysis (0.05 s^−1^) was very low, and the high catalytic efficiency was fully due to the extremely small apparent *K_M_* of ≈300 nM. Interestingly, when we used this value of *k_cat_* to derive the *K_M_* of isolated pentaglycine, we obtained *K_M_*= 22.7 mM. This value was in good agreement with the expected *K_M_* >> 4 mM and lay within the range of *K_M_* values observed for other enzymes.

The pentaglycine peptide within the staphylococcal peptidoglycan is attached to the stem peptides. In principle, the catalytic domain of lysostaphin can form favorable interactions with the stem peptide residues as well. Furthermore, pentaglycine incorporated into the cell wall might pre-exist in a conformation that favors interaction with lysostaphin, alleviating the inevitable entropy loss of the highly flexible pentaglycine molecule upon binding to the lysostaphin active site. Both these effects will result in an increased affinity of pentaglycine to the catalytic domain of lysostaphin and, thus, a lower value of *K_M_*. However, the increase in affinity due to these two factors alone is unlikely to be so dramatic.

Several previous works reported significantly different values of lysostaphin catalytic parameters. Warfield et al. [9] used pentaglycine labeled with a fluorophore and a quencher to analyze the lysostaphin activity in a FRET assay. They obtained *K_M_* of 200 or 300 µM, depending on the method of data analysis. Although the authors only reported *V_max_* of 63–76 pmol s^−1^ mg(Lst)^−1^ but not *k_cat_*, it can be calculated yielding the value of 0.0017–0.0021 s^−1^. The resulting catalytic efficiency *k_cat_*/*K_M_* of 7.0–8.7 M^−1^ s^−1^ is of the same order of magnitude as our results (2.2 M^−1^ s^−1^). Bardelang et al. [11] used a fluorescent protein with a pentaglycine linker and a covalently attached fluorescent dye as a substrate and obtained *K_M_* = 65 µM. The reported *V_max_* of 10.1 nM s^−1^ converted into *k_cat_* of 0.05 s^−1^, yielding *k_cat_*/*K_M_* = 777.0 M^−1^ s^−1^. However, in both works, pentaglycine was flanked by either fluorophores or bulky protein domains, which might have had an unpredictable impact on the enzyme activity on these substrates. Using unmodified pentaglycine peptide and detecting the hydrolysis products with NMR, Tossavainen et al. [14] obtained *k_cat_* of 0.006 s^−1^. This number, however, was inferred simply from the initial hydrolysis rate at a single substrate concentration of 1 mM and is likely an underestimation.

Despite these differences, all reports demonstrated the low turnover number and poor catalytic efficiency of lysostaphin on the isolated pentaglycine. These properties might have evolved to reduce the off-target cleavage and enzyme toxicity towards the producer cells, with the peptidoglycan-binding domain rescuing the lysostaphin activity towards its natural substrate—staphylococcal cell wall peptidoglycan.

## 3. Materials and Methods

### 3.1. Production and Purification of Recombinant Lysostaphin

Recombinant lysostaphin was produced essentially as described previously [16,17]. Briefly, *Escherichia coli* M15 (pRep4, pL330) was cultivated overnight in lysogeny broth (LB) medium supplemented with 25 µg mL^−1^ kanamycin and 150 µg mL^−1^ ampicillin. The overnight culture was used to inoculate fresh LB medium supplemented with the same antibiotics, and the cultures were grown for 3 h at 37 °C with 180 rpm shaking. The protein synthesis was induced by the addition of isopropyl β-d-1-thiogalactopyranoside (IPTG) to the final concentration of 0.5 mM and conducted for 3 h at 37 °C and 180 rpm. The cells were collected by centrifugation, resuspended in 25 mM Tris-HCl and 50 mM NaCl (pH 7.5), with 100 µg mL^−1^ lysozyme; incubated at ambient temperature for 30 min; and disrupted by sonication for 2 min at Bandelin Sonopuls HD3200 (Bandelin, Berlin, Germany) with 60% amplitude, 5 s pulses, 3 s intervals. The cell lysate was cleared by centrifugation and the supernatant was applied to the cation exchanger Unosphere S (Bio-Rad, Hercules, CA, USA) column equilibrated with 25 mM Tris-HCl and 50 mM NaCl (pH 7.5). The column was washed with the same buffer, and lysostaphin was eluted with 50–500 mM NaCl gradient in 20 mM Tris-HCl buffer (pH 7.5). The protein was dialyzed against 20 mM HEPES and 150 mM NaCl (pH 7.5), aliquoted, and stored at −80 °C.

To obtain lysostaphin variant with reduced enzymatic activity, we mixed lysostaphin with EDTA (final concentration 5 mM) and incubated the mixture at 37 °C for 3 h. Control lysostaphin was incubated at 37 °C for 3 h without EDTA.

### 3.2. Determination of Lysostaphin Enzymatic Activity

Ninhydrin (Dia-M, Moscow, Russia) was dissolved in DMSO at a 10% w/v concentration, aliquoted, and stored at −20 °C. Pentaglycine (sc-471644A, Santa Cruz Biotechnology, Inc., Dallas, TX, USA) was suspended in Milli-Q water at a concentration of 5 mM, dissolved by heating at 99 °C for 20 min on a water bath, aliquoted, and stored at −80 °C. Before the experiment, the aliquots of pentaglycine were thawed at 99 °C for 20 min in a dry block heater (Termit, DNA-Technology, Moscow, Russia) and cooled to ambient temperature. The reaction mixture consisting of 2, 3, or 4 mM pentaglycine and 20 mM HEPES (pH 7.5) was prepared, and the reaction was started by the addition of the appropriate amount of lysostaphin. The reaction mixture was divided into 20 µL aliquots in 0.5 mL Eppendorf tubes and incubated at 37 °C. The aliquots corresponding to the 0 h time point were immediately placed on ice and transferred to −80 °C. At defined time points, the aliquots were removed from the thermostat and placed at −80 °C to stop the reaction and preserve the samples for subsequent analysis. The collected samples were thawed at 99 °C for 10 min and 100 µL of ninhydrin working reagent (0.4% ninhydrin in 80% DMSO/20% water buffered with 20 mM HEPES (pH 7.5)) was added to the samples. The samples were mixed, incubated at 85 °C for 15 min, and cooled to ambient temperature. After that, 200 µL of water was added, the samples were mixed, 100 µL of the mixture was transferred to a 96-well plate, and the optical density at 595 nm was measured using an iMark microplate reader (Bio-Rad, Hercules, CA, USA). Pentaglycine integrity was not compromised by the repeated cycles of freezing and heating performed in the assay (Figure S3).

To convert the optical density into the concentration of unhydrolyzed pentaglycine, we conducted a calibration curve. Pentaglycine, diglycine, and triglycine (Sigma, St Louis, MO, USA) were mixed in appropriate proportions to simulate the process of pentaglycine hydrolysis, divided into 20 µL aliquots, and placed at −80 °C. The samples were thawed and processed as described above. Raw experimental data and conversion of optical density into pentaglycine concentration are provided in Supplementary Excel file.

### 3.3. Determination of Lysostaphin Bacteriolytic Activity

*Staphylococcus aureus* ATCC 29,213 cultivated on Brain Heart Infusion Agar (Sifin Diagnostics GmbH, Berlin, Germany) was inoculated into the medium containing 20 g L^−1^ peptone, 5 g L^−1^ NaCl, and 2.5 g L^−1^ K_2_HPO_4_ and grown at 37 °C and 110 rpm overnight. Staphylococcal cells were harvested by centrifugation at 4000× *g* for 5 min, resuspended in 20 mM HEPES and 150 mM NaCl (pH 7.5), centrifuged again at 4000× *g* for 5 min, and resuspended in the same buffer supplemented with 0.1% *w*/*v* bovine serum albumin to the desired optical density. The bacterial cell suspension was transferred to the flat-bottom 96-well plate (180 µL per well), 20 µL of 300 nM lysostaphin solution (final concentration 30 nM) in the same buffer was added to the plate wells, and the optical density at 550 nm was measured at 1 min intervals using Multiscan FC microplate reader equipped with a thermostat (Thermo Scientific, Waltham, MA, USA) at 37 °C with constant background shaking. Raw experimental data are provided in Supplementary Excel file.

### 3.4. Thin-Layer Chromatography

Thin-layer chromatography (TLC) was performed in a Sorbifill silica gel TLC plate (5–17 µm silica gel fraction, thickness 90–120 µm, Imid Ltd., Krasnodar, Russia) in the mixture of acetic acid, *n*-butanol, and water in 5:2:1 proportion. The reaction mixture samples were thawed at 99 °C for 10 min, cooled to the ambient temperature, and applied to the TLC plate in 5 consecutive 1 µL drops; the plate was allowed to dry between the application of each drop. After development, the TLC plate was dipped in a 0.2% ninhydrin solution in ethanol and heated at 99 °C to stain the separated peptides.

### 3.5. Data Analysis

To fit the Michaelis–Menten differential equation $\frac{d\left[S\right]}{dt}=-k_{cat}E_{0}\frac{\left[S\right]}{\left[S\right]+K_{M}}$ into the pentaglycine concentration vs. time data, we used a Python script (see MM_fit.ipynb, MM_fit.html in the Supplementary Materials).

To obtain *k_cat_*/*K_M_* parameters for pentaglycine cleavage using the simplified Michaelis–Menten equation, we transformed the differential equation $\frac{d\left[S\right]}{dt}=-E_{0}\frac{k_{cat}}{K_{M}}\left[S\right]$ into $\left[S\right]={\left[S\right]}_{0}\cdot e^{-E_{0}\frac{k_{cat}}{K_{M}}t}$, and Microsoft Excel Solver module was used to fit this equation into the pentaglycine concentration vs. time data.

To obtain *k_cat_* and *K_M_* parameters of lysostaphin on staphylococcal cells as substrate, we fitted the curves of staphylococcal cell suspension turbidity vs. time for different starting values of bacterial density with a 5-parameter logistic equation. To determine the rate of turbidity reduction in the linear portion of the curve, we identified the inflection point where the second derivative equaled zero, and the first derivative of the logistic curve at this point was calculated. Turbidity values were converted into the concentration of pentaglycine cross-bridges assuming the turbidity of 0.5 McFarland corresponding to 1.5 × 10^8^ cells mL^−1^ and 6.0 × 10^5^ pentaglycine cross-bridges per cell [15]. After that, *k_cat_* and *K_M_* parameters were estimated by fitting the equation $v=\frac{v_{max}\left[S\right]}{K_{M}+\left[S\right]}$ into the reaction rate vs. pentaglycine cross-bridge concentration plot. Calculations are provided in Supplementary Excel file.

Statistical analysis was performed using Microsoft Excel Data Analysis module.

## 4. Conclusions

In this work, we described a simple protocol for the determination of the enzymatic activity of lysostaphin. The protocol was based on the chromogenic reaction between peptide amino groups and ninhydrin. Unlike previously reported assays, the protocol only requires inexpensive, readily available reagents and basic laboratory equipment, allowing us to study the activity of lysostaphin on the unmodified pentaglycine peptide. The protocol can be used to investigate the effect of different treatments on lysostaphin activity, as well as to derive the catalytic parameters of lysostaphin on the isolated pentaglycine as a substrate, independently of the influence of the peptidoglycan-binding domain and the lysostaphin-induced cell lysis process. We further demonstrated the dramatic difference between the catalytic efficiencies of lysostaphin on the isolated pentaglycine peptide and whole staphylococcal cells, highlighting the relative contributions of enzymatic and peptidoglycan-binding domains to the cumulative bacteriolytic activity. In our opinion, deconvolution of the impact of the enzymatic and cell wall-binding domains is crucial for the understanding of the structure–activity relationships of antibacterial lysins and their rational design. The protocol described in this work will simplify this deconvolution for lysostaphin.

## Supplementary Materials

The following are available online https://www.mdpi.com/2079-6382/9/12/917/s1. Figure S1: Separation of oligoglycine peptides of different length with thin-layer chromatography. Figure S2: Comparison of the catalytic activity of control and EDTA-treated lysostaphin variants. Figure S3: Pentaglycine does not spontaneously hydrolyze upon repeated freezing and heating. Excel: raw experimental data and calculations; code.
