# Supplementary material for: A Simple Protocol for the Determination of Lysostaphin Enzymatic Activity
Source: Antibiotics (Basel). 2020 Dec 17;9(12):917. doi: 10.3390/antibiotics9120917 (PMC7766845; doi:10.3390/antibiotics9120917)
Supplement: Supplementary file 1 [file antibiotics-09-00917-s001.zip › MM_fit.html]

MM\_fit


To calculate the catalytic parameters of lysostaphin, 4 mM pentaglycine in 20 mM HEPES, pH 7.5, was mixed with 5 µM lysostaphin, incubated at 37°C for 0-48 h, and reacted with ninhydrin; results from 6 independent experiments were averaged, the optical density was converted into the pentaglycine concentration and fitted with the Michaelis-Menten differential equation. However, despite the overall good fit, the values of kcat and Km were unrealistically high. This was likely explained by the real Km being much higher than the initial substrate concentration (4 mM), making the individual values of kcat and Km indistinguishable since S/(Km+S) term of the Michaelis-Menten equation approaches S/Km at Km>>S.

In [1]:

```
import numpy
%matplotlib inline
from matplotlib import pyplot
from scipy.integrate import odeint
from scipy.optimize import minimize
```

In [2]:

```
# timepoints
time = [0., 4., 8., 24., 32., 48.]
# experimental substrate (pentaglycine) concentrations, mM
Sexp = [3.72528183,   3.37079727,   2.87016048,   1.66665636,
          1.05444409,   0.64184732]
```

In [3]:

```
# Michaelis-Menten differential equation
# dS/dt = -kcat*E0*(S/(Km+S))
def MM_de(t, kcat, km, E0, S0):
    de = lambda S, t: -kcat*E0*S/(km + S)
    return odeint(de, S0, t).flatten()
```

In [4]:

```
# the timeline with more timepoints than there are in the experiment
extended_time = numpy.arange(0,50,0.001)
# points in simulated data that correspond to the experimental points
indices = []
for point in time:
    indices.append(numpy.where(extended_time==point)[0][0])
# experimental setup
S0 = 4.0 # 4 mM pentaglycine
E0 = 0.005 # 5 uM lysostaphin
```

Fitting the Michaelis-Menten eqation into the data gives unrealistic values of kcat (3.8e14 per h) and Km (4.9e10 M).

In [5]:

```
# auxiliary minimization function
def _min(param):
    kcat, km = param
    p = MM_de(extended_time, kcat, km, E0, S0)
    pred = numpy.take(p, indices)
    result = numpy.power(pred-Sexp, 2).sum()
    return result

def plot(param):
    kcat, km = param
    p = MM_de(extended_time, kcat, km, E0, S0)
    pyplot.plot(time, Sexp)
    pyplot.plot(extended_time, p)
    pyplot.show()

# minimization routine
result = minimize(_min, x0=[1000., 100.], method='Nelder-Mead')
print "kcat = %2.1e, Km = %2.1e, kcat/Km = %d" % (result.x[0], result.x[1], result.x[0]/result.x[1])

pyplot.plot(extended_time, MM_de(extended_time, result.x[0], result.x[1], E0, S0))
pyplot.plot(time, Sexp)
pyplot.show()
```

```
kcat = 3.8e+14, Km = 4.9e+13, kcat/Km = 7
```
