# Supplementary material for: A Simple Protocol for the Determination of Lysostaphin Enzymatic Activity
Source: Antibiotics (Basel). 2020 Dec 17;9(12):917. doi: 10.3390/antibiotics9120917 (PMC7766845; doi:10.3390/antibiotics9120917)
Supplement: Supplementary file 1 [file antibiotics-09-00917-s001.zip › Supplementary_figures.pdf]

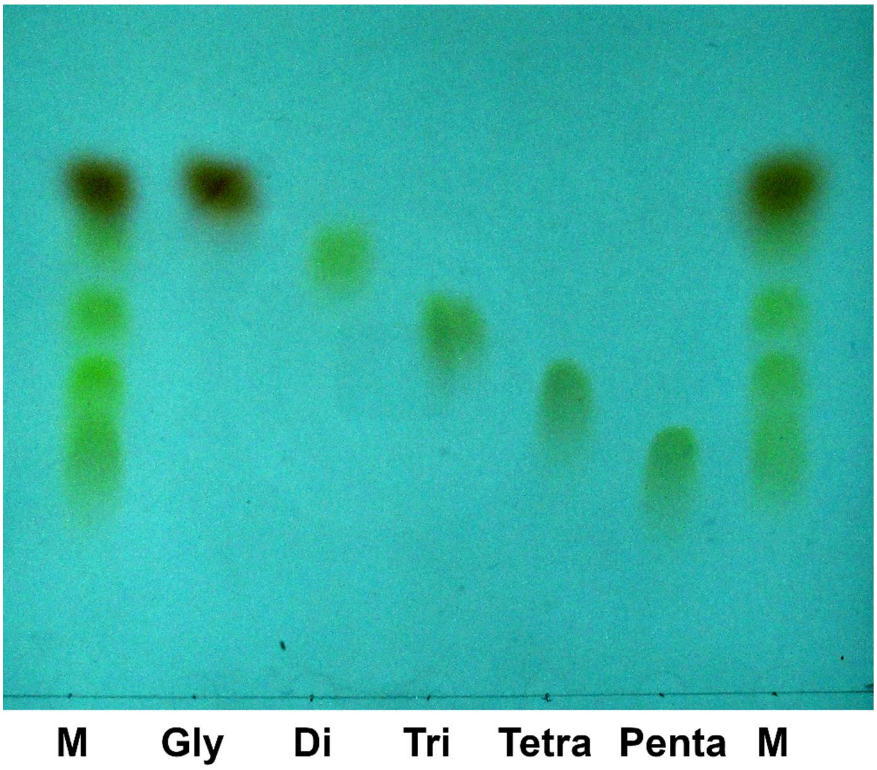

Figure S1. Separation of oligoglycine peptides of different length with thin layer chromatography. M – mixture of five oligoglycines; Gly – glicyne; Di – diglycine; Tri – triglycine; Tetra – tetraglycine; Penta – pentaglycine.

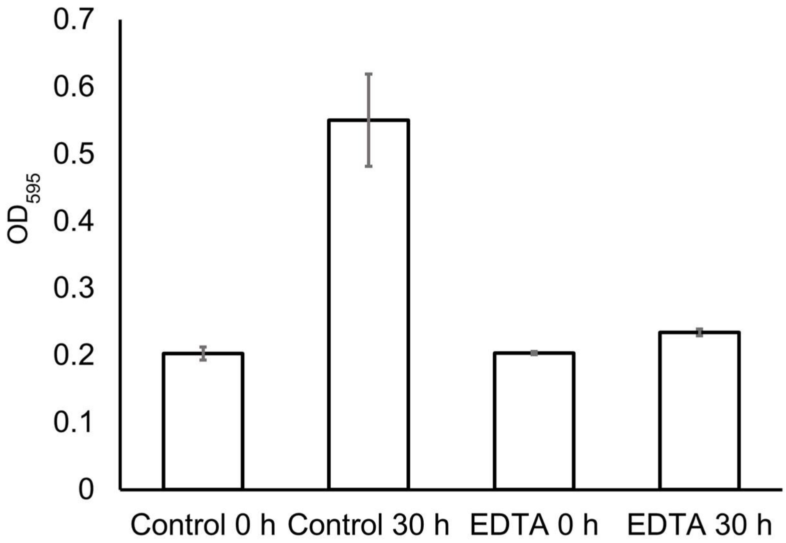

Figure S2. Comparison of the catalytic activity of control and EDTA-treated lysostaphin variants. 4 mM pentaglycine in 20 mM HEPES, pH 7.5, was mixed with either 3 μM lysostaphin (control), or 3 μM lysostaphin treated with 5 mM EDTA for 3 h, incubated at 37°C for 30 h, and reacted with ninhydrin; the average results from 3 independent experiments are shown, error bars represent standard deviation.

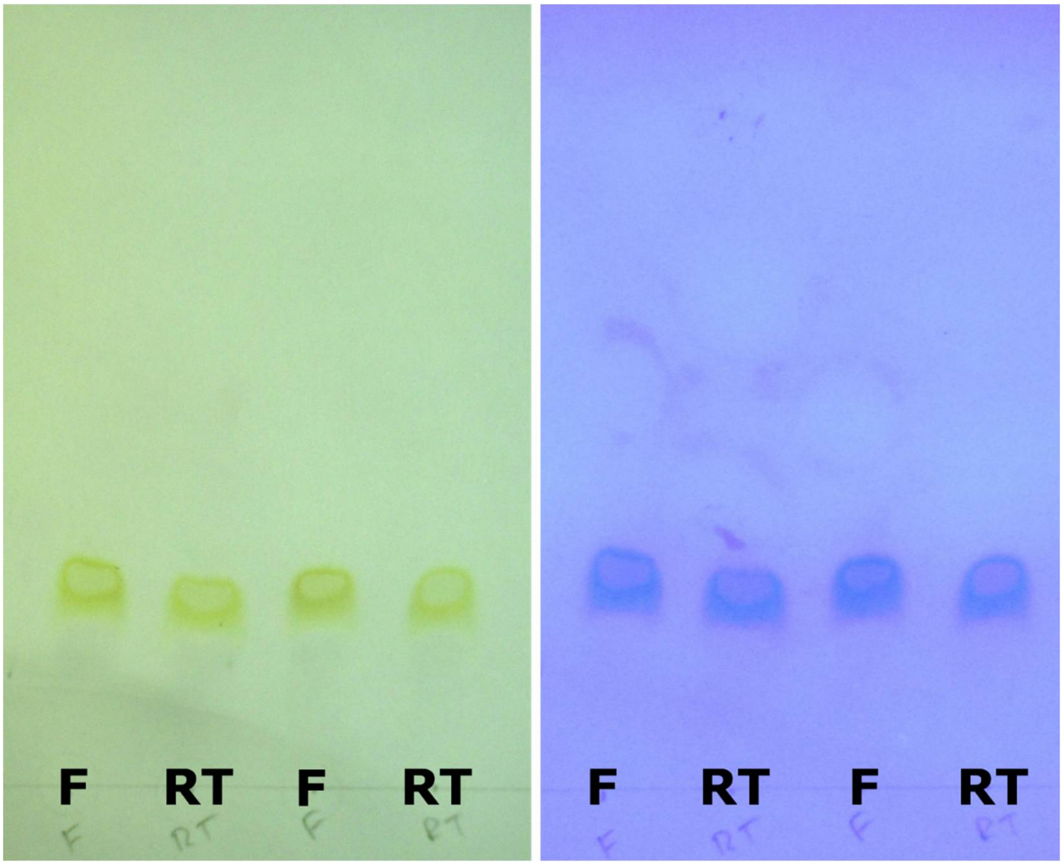

Figure S3. Pentaglycine does not spontaneously hydrolyze upon repeated freezing and heating. Pentaglycine was dissolved in milliQ water in the concentration of 5 mM by heating at 99°C for 20 min. The solution was divided into two parts. The first part was frozen at -80°C, thawed by heating at 99°C for 20 min, diluted to 4 mM, buffered with 20 mM HEPES, pH 7.5, aliquoted into 20 μL aliquots, frozen at -80°C, and thawed by heating at 99°C for 10 min, thus simulating the freeze/heat cycles pentaglycine undergoes during the protocol (F – "full protocol" variant). The second part was diluted to 4 mM, buffered with 20 mM HEPES, pH 7.5, aliquoted into 20 μL aliquots, and kept at room temperature (RT – "room temperature" variant). Both parts were applied to a TLC plate and developed in 2:5:1 butanol: acetic acid: water mixture. The plate was stained with ninhydrin and visualized under white light (left) or UV (right). No traces of spontaneous hydrolysis are seen in either pentaglycine variant.
